# Glycosaminoglycans and Proteoglycans

**DOI:** 10.3390/ph11010027

**Published:** 2018-02-27

**Authors:** Vitor H. Pomin, Barbara Mulloy

**Affiliations:** 1 Program of Glycobiology, Institute of Medical Biochemistry Leopoldo de Meis and University Hospital Clementino Fraga Filho, Federal University of Rio de Janeiro, Rio de Janeiro, RJ 21941-913, Brazil; pominvh@bioqmed.ufrj.br; 2 Glycosciences Laboratory, Department of Medicine, Imperial College London, Burlington Danes Building, Du Cane Road, London W12 0NN, UK

**Keywords:** chondroitin sulfate, decorin, dermatan sulfate, glycosaminoglycans, glypican, heparan sulfate, heparin, hyaluronan, keratan sulfate, perlecan, proteoglycans, serglycin, syndecan

## Abstract

In this editorial to MDPI *Pharmaceuticals* special issue “*Glycosaminoglycans and Proteoglycans*” we describe in outline the common structural features of glycosaminoglycans and the characteristics of proteoglycans, including the intracellular proteoglycan, serglycin, cell-surface proteoglycans, like syndecans and glypicans, and the extracellular matrix proteoglycans, like aggrecan, perlecan, and small leucine-rich proteoglycans. The context in which the pharmaceutical uses of glycosaminoglycans and proteoglycans are presented in this special issue is given at the very end.

## 1. Introduction

This short article is intended to provide a brief introduction to the structures of glycosaminoglycans (GAGs) and proteoglycans (PGs) to set the articles in this special issue of *Pharmaceuticals* on “*Proteoglycans and Glycosaminoglycans*” into context. The class of glycosylated proteins known as PGs is represented in the pharmaceutical world chiefly by its carbohydrate constituents. These are polysaccharides known as GAGs, such as heparin (Hp) [1] and chondroitin sulfate (CS) [2]. When attached to their native protein cores these polysaccharides form the glycoconjugates known as PGs. Whole PGs are less often proposed as therapeutic agents, though recently, particularly in the context of regenerative medicine, the concept of PG mimetics, ‘neoproteoglycans’, is becoming more familiar [3]. The development of PG- and GAG-based medicines is beginning to take into account the way the GAGs are organized and presented by attachment to their PG cores, as well as the sequences and covalent structures of the compounds themselves. In this special issue, contributed articles will cover the current pharmaceutical uses of GAGs and their mimetics, with others describing the involvement of GAGs in processes, such as cell growth and differentiation, morphogenesis, inflammation, and healing; all of which are likely to give rise to future therapeutic uses of GAGs and PGs.

## 2. Glycosaminoglycans

### 2.1. Structural Features

GAGs are linear and heterogeneous sulfated glycans. Although they are structurally complex, the backbones of these polysaccharides are simply made up of repeating disaccharide building blocks composed of alternating uronic acid (UA) and hexosamine units. The UA units can be either β-d-glucuronic acid (GlcA) or its C5 epimerized version, α-l-iduronic acid (IdoA). The amino sugars can be either glucose (Glc)-based (α-d- or β-d-glucosamine, GlcN) or galactose (Gal)-based, as *N*-acetyl-β-d-galactosamine (GalNAc). The permutation of these monosaccharide units within the GAG backbones gives rise to different GAG families, such as the GlcN-containing heparan sulfate (HS) and Hp [4,5], and the GalNAc-containing CS and dermatan sulfate (DS) [6]. Keratan sulfate (KS) alternates *N*-acetyl-glucosamine (GlcNAc) with Gal, and does not contain UA [7,8]; hyaluronan or hyaluronic acid (HA) alternates GlcNAc with GlcA, and does not have a protein core [9].

**Figure 1:**
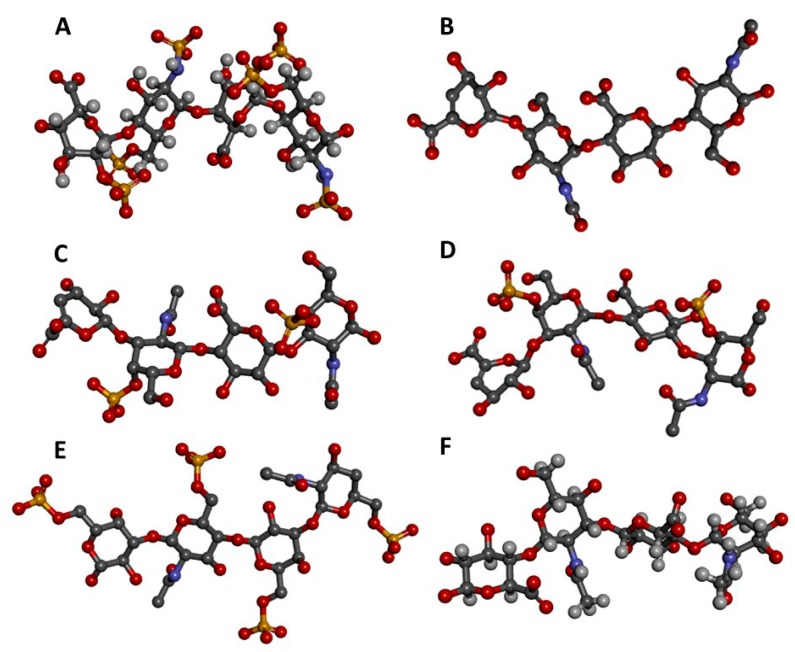
Three-dimensional tetrasaccharide representations, taken from files in the PDB as indicated, of: (**A**) Heparin [IdoA2S(α1→4)GlcNS6S(α1→4)IdoA2S(α1→4)GlcNS6S] from 1HPN; (**B**) heparan sulfate [GlcA(β1→4)GlcNAc(α1→4)GlcA(β1→4)GlcNAc] from 3E7J; (**C**) chondroitin 4-sulfate [GlcA(β1→3)GalNAc4S(β1→4)GlcA(β1→3)GalNAc4S] from 1OFM; (**D**) dermatan sulfate [IdoA(α1→3)GalNAc4S(β1→4)IdoA(α1→3)GalNAc4S] from 1OFL; (**E**) keratan sulfate [Gal6S(β1→4)GlcNAc6S(β1→3)Gal6S(β1→4)GlcNAc6S] from 1KES; and (**F**) hyaluronan [GlcA(β1→3)GlcNAc(β1→4)GlcA(β1→3)GlcNAc] from 2BVK. The atoms in the ball-stick representations are carbon (grey), nitrogen (blue), hydrogen (light grey); oxygen (red) and sulfur (yellow). A and F are NMR solution structures, and non-exchangeable protons are shown; the others are crystal structures so are shown without protons.

The disaccharides of HS and Hp are both composed of alternating 4-linked UA and 4-linked α-GlcN units [4,5]. Structurally, HS and Hp differ only in the relative proportions of their monosaccharide and disaccharide substructures. HS has *β*-d-GlcA as its major UA type while Hp has α-l-IdoA. GlcA in HS usually alternates with GlcNAc units, but lower amounts of *N*-sulfated glucosamine (GlcNS) and rare amounts of unsubstituted GlcN can also occur. Hp is conversely predominantly composed of 2-sulfated IdoA units (IdoA2S) together with *N*,6-di-sulfated GlcN units (GlcNS6S). These disaccharides are reflected in the three-dimensional structures of HS/Hp tetrasaccharides shown in Figure 1A,B. They are experimental structures from the Protein Databank (PDB), and clearly adopt fairly linear shapes, though in solution they may twist and fold to varying extents depending on the exact monosaccharide sequence [10].

Rarely, 3-*O*-sulfation at the GlcNS6S units can also occur (GlcNS3S6S) in both HS and Hp but is more common in the Hp chains; a pentasaccharide containing this unusual monosaccharide residue confers high affinity for antithrombin and, thereby, high anticoagulant activity, on Hp [1].

Since the length of GAG chains can vary widely, both among different GAG types and among chains of the same GAG type, the range of MWs in GAGs is very broad, ranging from a few kDa to over a hundred kDa. It is also true that manufacturing processes can alter the molecular weight (MW) of GAGs; for example, unfractionated heparin (UFH) and low-molecular weight heparin (LMWH) have chains whose average MWs are respectively ~15 kDa (~25 disaccharide units) and less than 8 kDa (~12 disaccharide units or fewer). The measurement of MWs for LMWH is not straightforward, as Hp consists of a mixture of sequence types as described above, each occurring in domains with particular hydrodynamic and conformational properties. The contribution of Jian Liu and co-workers, introducing the concept of homogeneous structurally defined MW markers for Hp is a real step forward [11].

CS disaccharides are essentially composed of alternating 4-linked β-d-GlcA and 3-linked GalNAc units. Various CS subtypes exist; for example, CS-A is mostly 4-sulfated at the GalNAc units (Figure 1C), while CS-C is predominantly 6-sulfated. CS-B, widely known as DS (Figure 1D), has α-l-IdoA units rather than β-d-GlcA. The IdoA units in DS may occasionally bear 2-sulfation while the GalNAc units are mostly 4-sulfated [6]. The MWs of CS-A, CS-B, and CS-C are around 20–60 kDa. Thus, approximately 35–130 disaccharide units for CS-A, CS-B, and CS-C calculated based on the MWs of their commonest disaccharide structures.

KS disaccharides are composed of alternating 3-linked β-d-Gal and 4-linked β-d-GlcNAc units [8]. These KS disaccharides can bear sulfation at the 6-position of either unit (Figure 1E), although sulfation at GlcNAc occurs more often [7,8]. The typical average MW of KS is around 20 kDa, thus, around 45 disaccharide units assuming the MW of the commonest structure. HA is the only GAG type which is not sulfated; it is composed of repeating disaccharide units of alternating 4-linked β-d-GlcA and 3-linked β-d-GlcNAc (Figure 1F) [9], and has the longest chain among all GAG types. The MW of HA is usually above 100 kDa and the degree of polymerization of HA is, therefore, in the range of at least 255 disaccharide units/chain, ranging upwards to MW of several million [12]. This very high MW polysaccharide has high viscosity at low concentration [12].

**Figure 2:**
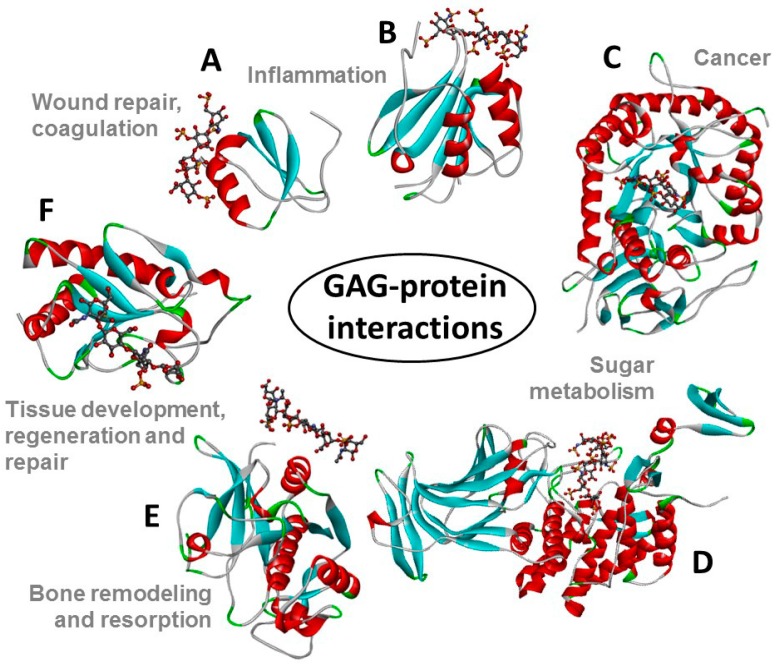
Structural representations from the crystal structures of some illustrative GAG-protein complexes in PDB: (**A**) macrophage inflammatory protein 1-alpha (CCL3) monomer + Hp 4-mer (from 5D65); (**B**) platelet factor 4 (CXCL4) dimer + fondaparinux (antithrombin-high affinity Hp 5-mer) (from 4R9W); (**C**) human heparanase complex + Hp tetrasaccharide (from 5E9C); (**D**) d-glucuronyl C5-epimerase + Hp 6-mer (from 4PXQ); (**E**) cathepsin K monomer + DS 6-mer (from 4N79); and (**F**) Sonic Hedgehog (Shh) monomer + CS-A 4-mer (from 4C4M). The atoms of the GAG ligands represented in the ball-stick view are carbon (grey), nitrogen (blue), hydrogen (light grey); oxygen (red) and sulfur (yellow). In the proteins, the alpha-helices, beta-sheets, loops, and random coils are represented, respectively, in red, blue, green and grey. The pathophysiological systems in which these complexes play a role are indicated by grey fonts in the panel.

The structures of all these GAG families are represented in Figure 1. The GAG structural variations and heterogeneities associated with their high sulfation content (except HA) and their common occurrence at the extracellular matrix (ECM) or at the surface of cells are all contributing factors to the diversity of their biomedical roles because they give GAGs the capacity of binding to multiple extracellular proteins [13,14] whose actions are spread in various pathophysiological events (Figure 2).

### 2.2. Pharmaceutical Applications of GAGs and PGs

GAGs interact with proteins in many biological systems, and as a consequence they have numerous biological and therapeutic functions [15,16,17,18,19,20,21]. In fact, GAGs can be considered the most exploited carbohydrates in the pharmaceutical market [14]. The use of GAGs as therapeutic agents is dominated by the potent anticoagulant and antithrombotic GAG Hp [1], isolated from mast-cell rich tissues such as intestinal mucosa of pigs and cattle [22]. Hp is the by far the most widely used GAG type and perhaps the most common therapeutic carbohydrate worldwide [23]; it is used in treatments and prophylaxis of thromboembolic disorders [1,24,25]. The pharmaceutical analysis of Hp preparations is rarely simple; in this issue, a radical new approach to molecular weight measurements using synthetic calibrant and computational extrapolation [11] is proposed. The potent anticoagulant activity of Hp occasionally requires an antidote in clinical use. Hogwood and co-workers address the use of protamine to neutralize Hp from different species and tissues [26]. The heterogeneity of Hp, and other consequences of its origins as a natural mammalian product, has led to the development of synthetic and semi-synthetic Hp mimetics, as described by Mohamed and Coombe in this issue [27].

CS [2] and HA [28] are also exploited as pharmaceutical ingredients. CS can be used as an alternative therapeutic in cases of osteoarthritis [29], and sometimes even osteoporosis [30], because of its essential roles in cartilage and other connective tissues, though some degree of caution in interpretation of trials may be wise [30,31]. The beneficial use of CS in arthritic disease is usually associated with the use of GlcN, another key constituent of cartilage tissues. Santos and coworkers have in this issue described a systematic process based on electrophoresis, liquid-chromatography and NMR for assessment and control of pharmaceutical preparations of CS combined with GlcN [32]. KS can be employed as active ingredient in eye drops for treatment of certain visual dysfunctions; KS is one of principal functional components in cornea [7,8]. The best-known physicochemical property of HA is its capacity of forming gels in solution. This property enables HA to be used as a vehicle to make specific hydrogel formulations for regenerative medicine [33,34]. HA-based medium can be employed in cosmetics to soften and smooth skin owing to its inherent regenerative and hydrating properties; HA is an important functional component of the ECM of skin [35,36].

## 3. Proteoglycans

There are fewer than 50 distinct PG genes, though many more proteins due to alternative splicing [37]. Fewer than 20 distinct mammalian HS-PG core proteins have so far been identified [38]. This apparently limited repertoire of structures is responsible for numerous structural and functional properties of animal cells and ECM. Though many PGs are also glycoproteins, bearing *N*- and *O*-glycans, the defining type of glycosylation for PGs is the presence of one or more—sometimes many more—*O*-linked GAG chains. These GAGs make their contribution to the biological functions of PGs in many ways, and in some extreme cases the protein core may simply act as a scaffold for the presentation of the biologically-active GAG.

Most PGs act predominantly in the extracellular space, either as structural elements or as ligands for the many small protein growth factors, cytokines, chemokines, and morphogens that regulate embryonic development, inflammatory responses to pathogens and injury, and communication between cells [16,37]. PGs are often large proteins, heavily glycosylated and attached to membranes at the cell surface or in the ECM.

**Figure 3:**
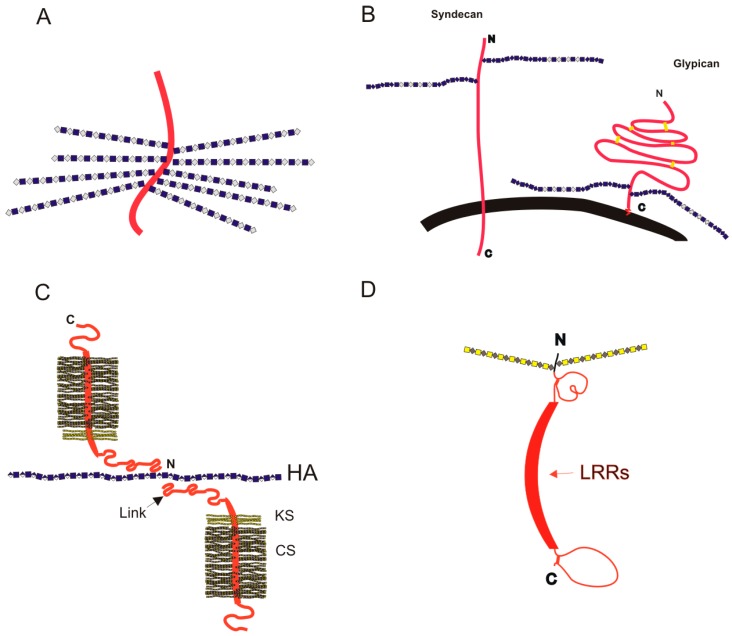
Schematic cartoon images (not to scale) for PGs on the cell surface and in the ECM. Protein chains are shown as red ribbons, and GAG chains are depicted in a simplified form of the symbology recommended by the Consortium for Structural Glycomics [40]; (**A**) the intracellular PG serglycin, bearing closely packed Hp (or oversulfated chondroitin) chains, on a small peptide core; (**B**) cell-surface PGs syndecan and glypican; the cell membrane is shown in black; (**C**) the complex between aggrecan and HA, mediated by Link protein, that forms the structural basis for cartilage elasticity; and (**D**) a generic diagram of a SLRP, such as biglycan or decorin. Between the globular regions near the N- and C-termini the leucine-rich repeats (LRRs) form a curved structure; the dimers can form by interaction between the two offset concave faces of monomers.

Descriptions of PGs have often used simple schematic diagrams, such as those shown in Figure 3 and in [39] depicting cartoon schematics for two major cell-surface PGs, syndecan, and glypican, and several PGs of the ECM (aggrecan and the small leucine rich PGs (SLRPs) such as decorin, lumican and biglycan). They are useful to show the rough relative sizes of PGs, with number and approximate attachment sites of GAG chains. These simple diagrams can give a clue as to function; where GAG chains are closely spaced they are likely to have a space-packing function, in the extracellular matrix in the case of aggrecan, and in the mast cell secretory granules in the case of serglycin. Where the GAG chains are less dense, such as on the cell surface attached to glypican or syndecan, their role is more likely to be in signaling, or in tissue organization. In this short article we will introduce only a few of the best known PGs; more comprehensive descriptions can be found elsewhere [37].

### 3.1. Intracellular Proteoglycans

The PG from which Hp is derived is called serglycin [41], and it is unusual as a PG in that it is found only in the granules of mast cells and related cell types, not, like other PGs, in the ECM or on the cell surface. The narrowly spaced Hp chains on the small peptide backbone of serglycin accommodate positively charged proteins, such as the proteolytic enzymes found in mast cell secretory granules, allowing much closer packing by neutralizing their charge [42]. Hp is partially depolymerized on degranulation of mast cells, to give the MW distributions characteristic of Hp sodium in clinical use [43]. Further deliberate depolymerization gives rise to the range of LMWH products, such as enoxaparin, described by Arnold et al. in this issue [11].

### 3.2. Cell Surface PGs: Syndecans and Glypicans

The cell-surface PG groups of glypicans are modulators of morphogens such as Wnt, bone morphogenetic proteins (BMPs), fibroblast growth factors (FGFs) and Sonic Hedgehog (Shh) [44]. There are six mammalian glypican genes, giving rise to core proteins glypican-1 to glypican-6, all of them attached to the outer surface of the cell membrane by a C-terminal glycosylphosphatidylinositol (GPI) anchor [44]. The ‘cartoon’ schematics shown in Figure 3 depict the glypicans as globular proteins, with an extended sequence containing HS attachment sites between the globular region of the protein and the GPI anchor [45]. The glypican-1 protein measures about 120 Å along its long axis [46], whereas a 50–100 residue long HS chain could extend to 200–400 Å. The HS attachment site is a few amino acid residues away from the globular protein core, with more than 50 amino acids between it and the GPI anchor, allowing considerable freedom in the orientation of protein and GAG [46], both of which may be available for interactions with extracellular proteins simultaneously.

By contrast, the four mammalian syndecans [45,47] hold their GAG side-chains out further from the cell surface, around 200 amino acids from the cell surface, though we have no information on the 3D structure of syndecan ectodomains. Syndecans have a transmembrane domain, and a cytodomain that interacts with kinases and the actin cytoskeleton [47].

Cell-surface GAGS attached to these PGs play a part in the early stages of viral invasion of the host cell. As an example, Kim and co-workers in this issue describe the recognition of cell surface GAGs by the envelope proteins of Flaviviruses, including dengue and yellow fever viruses [17]. In addition, viral carbohydrates are involved in interaction with the cell surface receptor dendritic cell-specific ICAM-grabbing non-integrin, DC-SIGN [17].

GAGs are also involved in the cell uptake of potentially useful drug-delivery systems. Takechi-Haraya and associates have in this issue described the contribution of GAGs in the non-endocytic direct cell membrane translocation of arginine-rich cell-penetrating peptides by studying the cell-permeation of octaarginine monitored through real-time in-cell ^19^F NMR spectroscopy [21].

Damage to the cell surface glycocalyx, of which GAGs form an important part, is a feature of disease states of the vascular endothelium. Wodicka and coworkers have developed a PG mimetic designed to bind to inflamed endothelium and prevent platelet binding to create a more quiescent endothelial state [20].

### 3.3. Extracellular Matrix Proteoglycans

#### 3.3.1. Aggrecan in the Extracellular Matrix

The most abundant PG in ECM rich tissues such as cartilage is aggrecan, which forms very large aggregates with HA, mediated by the link protein [18]. These aggregates are held in a collagen network to form strong but elastic articular cartilage structure, a target tissue for regenerative medicine due to the ubiquity of osteoarthritis in the aging population [18]. The close packing of CS chains attached to the aggrecan core gives the molecule a ‘bottle-brush’ appearance, not only in the simplified diagram in Figure 3C, but also in atomic force microscopy images [48].

#### 3.3.2. Perlecan at the Basement Membrane

No 3D structure has been published for the large, multi-domain PG perlecan, found at the basement membrane and in the pericellular space. As an HS-bearing PG in the space between cells, perlecan can act as an extracellular storage device for growth factors such as FGFs and is involved in angiogenesis through its interactions with vascular endothelial growth factor (VEGF) [49].

#### 3.3.3. Small Leucine-Rich Proteoglycans

The SLRPs exemplified by decorin have ordering functions in tissues; decorin in tendons appears to wrap round the D-band of collagen fibrils forming a ring-mesh of GAG [50]. SLRPs are also important for the structure of cornea, for which transparency requires an absolutely regular structure. The SLRP lumican is substituted with three *N*-linked KS chains [51] and its absence leads to a loss of order in the array of collagen fibrils, resulting in opacity of the cornea [52]. The importance of the SLRPs and their DS chains provides the focus of Mizumoto and coworkers, who, in this issue, have described mutations in human genes encoding the glycosyltransferases, epimerases, and sulfotransferases responsible for the biosynthesis of DS chains and their effects on connective tissue disorders, including some forms of Ehlers-Danlos syndrome [19].

## 4. The Context of Glycosaminoglycans, Proteoglycans, and Their Pharmaceutical Uses in the Special Issue “*Glycosaminoglycans and Proteoglycans*”

This special issue on “*Glycosaminoglycans and Proteoglycans*” contains a diverse collection of articles, illustrating the range of biological systems in which GAGs and PGs operate and can be considered in the design of pharmaceutical interventions. They range from the analysis of GAG pharmaceutical products [32] and Hp neutralization [26] through Hp mimetics [27], GAG-mediated uptake of cell penetrating peptides [21], to the development of PG mimics for endothelial repair [20]. Review articles cast a new light on regenerative medicine [18], chemokine-GAG interactions [16], the DS-PG related human genetic disorders, and Flavivirus interactions with host cells [17]. 

Some puzzles, vital to the progress of GAG and PG pharmaceuticals research, remain. The complex substitution patterns that GAGs are capable of displaying allow a remarkably large repertoire of structural motifs for recognition by proteins at the cell surface and between cells. We know that HS epitopes vary through processes of stem cell differentiation and embryonic development [18], and that the fine structure of GAGs adds to the intricacies of interactions between chemokines and their receptors [16]. We still, however, have no clear strategy to determine the exact preference of a protein for a specific sequence within a GAG polysaccharide chain, as we are hampered by a lack of experimental tools for the task. The development of synthetic GAG mimetics [27] may, in the future, offer libraries of homogenous GAG-like compounds that will allow detailed identification of protein ligand motifs within long GAG chains, leading to the possibility of the rational design of a whole new class of pharmaceuticals.
